# Beta-blocker use and outcome after allogeneic hematopoietic stem cell transplantation in acute myeloid leukemia

**DOI:** 10.1007/s00277-026-06962-w

**Published:** 2026-03-25

**Authors:** Johanna Werk, David Baden, Inna Shaforostova, Jan-Henrik Mikesch, Christian Reicherts, Julia Marx, Georg Lenz, Christoph Schliemann, Matthias Stelljes, Alexander Pohlmann

**Affiliations:** 1 Department of Medicine A, University Hospital Münster, Albert-Schweitzer-Campus 1, Münster, 48149 Germany; 2 Department of Medicine II, University Hospital Schleswig-Holstein, Lübeck, Germany; 3 Department of Medical Oncology, Inselspital, Bern University Hospital, Bern, 3010 Switzerland

**Keywords:** Acute myeloid leukemia, Allogeneic hematopoietic stem cell transplantation, Beta-blocker, Non-relapse mortality, Graft-versus-leukemia

## Abstract

Allogeneic hematopoietic stem cell transplantation (HSCT) is curative for patients with acute myeloid leukemia (AML) through an immune-mediated graft-versus-leukemia effect, but relapse is common. Studies in solid tumors have shown that adrenergic stress impairs anti-tumor immunity, and that beta-blockade can prolong survival in mice and patients receiving immunotherapy. To this end, we investigated whether beta-blocker use impacts outcomes in AML patients undergoing HSCT. We analyzed the cumulative incidences of relapse (CIR), non-relapse mortality (NRM) and overall survival (OS) in 413 patients with AML who underwent first allogeneic HSCT. A total of 112 patients (27%) received beta-blockers after HSCT. The use of beta-blockers was associated with a lower CIR (*P* = 0.024), but with a higher NRM (*P* = 0.002); OS did not differ. In multivariable analyses, beta-blocker use emerged as an independent factor associated with CIR (*P* = 0.024) and NRM (*P* = 0.012), but not OS (*P* = 0.353). The occurrence of acute or chronic graft-versus-host disease (GvHD) was not significantly associated with beta-blocker use, but chronic GvHD or organ toxicity was more often the primary cause of death in the beta-blocker group (*P* = 0.041). The use of angiotensin-converting enzyme (ACE) inhibitors/angiotensin II receptor blockers (ARBs) or calcium channel blockers was not significantly associated with outcome. Our study is the first to demonstrate an association between beta-blocker use and post-transplant outcome in AML and supports further investigation of carefully titrated beta-adrenergic blockade as a potential means to influence donor cell immune responses in the transplant setting.

**Supplementary Information:**

The online version contains supplementary material available at 10.1007/s00277-026-06962-w.

## Introduction

Allogeneic hematopoietic stem cell transplantation (HSCT) remains a curative treatment approach for patients with acute myeloid leukemia (AML). Despite improvements in supportive care and transplant procedures, long-term survival is still compromised by relapse, reported as cumulative incidence of relapse (CIR), and non-relapse mortality (NRM) [1]. Strategies to identify modifiable risk factors are therefore of major clinical interest.

Adrenergic signaling has been shown to regulate tumor biology and immune responses. Preclinical models demonstrated that stress hormones can promote tumor growth, metastasis, and immune dysregulation [2–4]. These findings provide a rationale for investigating pharmacological beta-adrenergic blockade as a potential modifier of cancer outcomes.

Clinically, evidence remains heterogeneous. Some retrospective studies suggested favorable effects of beta-blocker therapy on tumor progression and survival in solid tumors [5]. However, results across different entities and treatment settings have been inconsistent. A recent systematic review and meta-analysis, comprising 79 studies with nearly 500,000 patients, reported an association between beta-blocker use and improved progression-free survival but no consistent benefit for overall or cancer-specific survival. Importantly, substantial heterogeneity and risk of bias were noted [6].

To date, no study has investigated the potential influence of beta-blocker exposure on outcomes after HSCT in patients with AML. The present analysis therefore aimed to evaluate the association between beta-blocker use and post-transplant outcomes including cumulative incidence of relapse (CIR), non-relapse mortality (NRM), and overall survival (OS) in AML patients undergoing allogeneic HSCT.

## Methods

### Patient characteristics

**Fig. 1:**
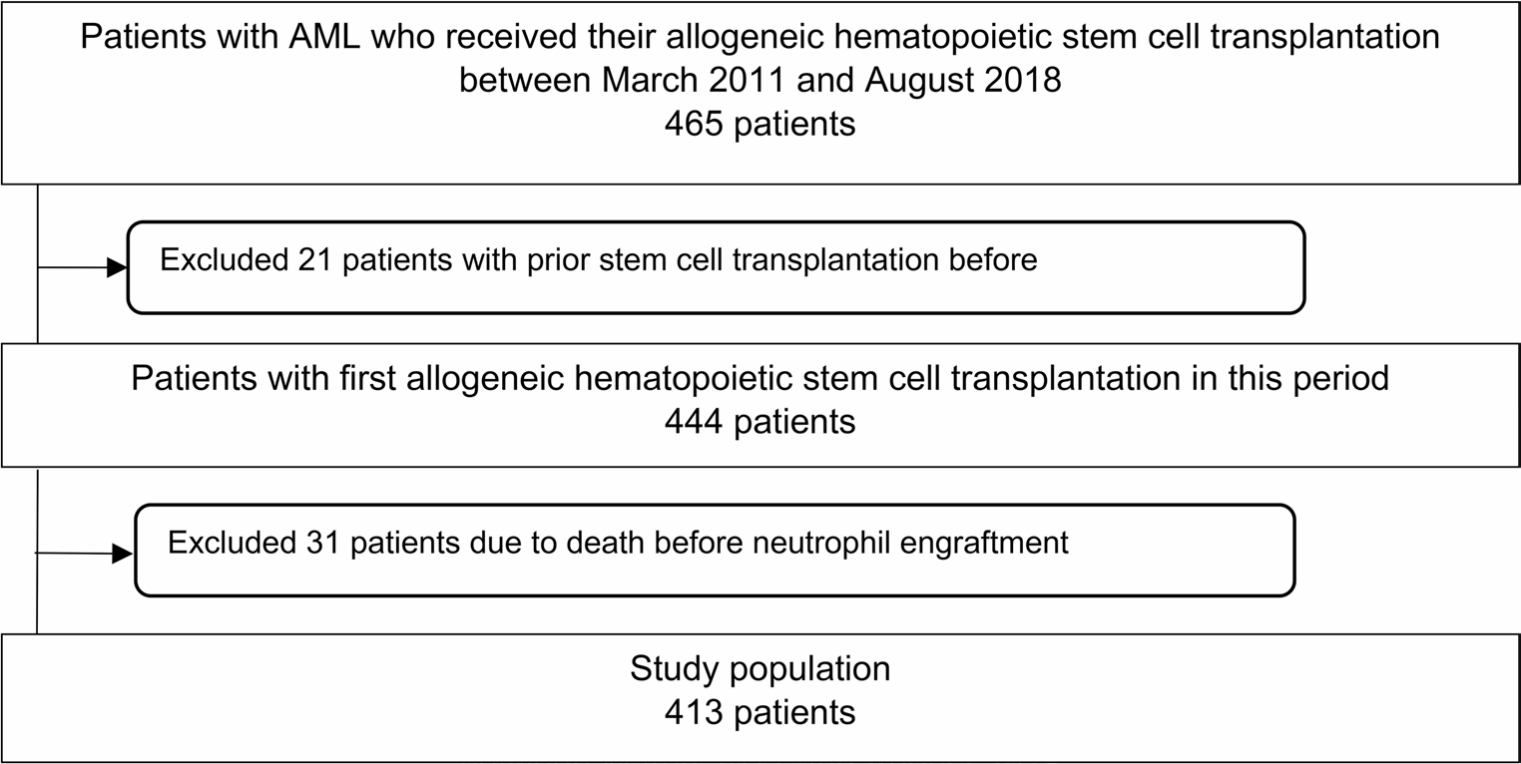
Flow diagram showing inclusion and exclusion of patients with acute myeloid leukemia (AML) undergoing allogeneic hematopoietic stem cell transplantation (HSCT) between March 2011 and August 2018. A total of 413 patients were included in the final analysis

Patients with AML who underwent first allogeneic HSCT at the Bone Marrow Transplantation Center of the University Hospital Münster, Germany, between March 2011 and August 2018 were included. Initially, 465 transplanted AML patients were screened, of whom 52 patients were excluded. Twenty-one patients were excluded because of previous HSCT (e.g. for myelodysplastic syndrome) and 31 patients were excluded because of early death after transplantation before neutrophil engraftment. Thus, a total of 413 AML patients were included in the final analysis set (Fig. 1).

The intervention of interest was beta-blocker use, defined as (1) beta-blockers as part of the discharge medication after allogeneic HSCT or (2) beta-blocker medication for at least 14 consecutive days after neutrophil engraftment during the inpatient stay of HSCT. All patients taking beta-blockers at discharge had to continue taking beta-blockers for at least 60 days after HSCT. We therefore applied a landmark design including only patients who survived until neutrophil engraftment; exposure to beta-blockers was defined thereafter. Beta-blockers were prescribed for standard clinical indications at the treating physician’s discretion; no patient received beta-blockers with the intent to influence relapse or other study endpoints. Patients with short-term beta-blocker exposure not meeting the predefined criteria were classified as non-users (*n* = 25). Beta-blocker exposure was observed throughout the entire study period (2011–2018) without clustering in a specific time window (Supplementary Table 4). In addition, patients taking beta-blockers were further divided into high-dose and low-dose beta-blocker groups based on total daily dose. The low-dose group included those patients with a daily dose of less than or equal to 30 mg propranolol, 5 mg bisoprolol, 70 mg metoprolol, 2.5 mg nebivolol or 12.5 mg carvedilol. Any daily dose above these limits was considered high-dose. We also recorded the intake of calcium channel blockers, angiotensin-converting enzyme (ACE) inhibitors and angiotensin II receptor blockers (ARBs).

The study was approved by the Ethics Committee of Westphalia-Lippe (2021-490-f-S) and was conducted in accordance with the Declaration of Helsinki and its later amendments.

#### Outcomes and measurements

Patient data were collected retrospectively from medical records. Post-transplant AML relapse was defined as at least 5% leukemic blasts in a bone marrow aspirate, reappearance of blasts in peripheral blood or new extramedullary leukemia. The CIR was calculated by considering deaths from causes other than relapse as a competing risk. Deaths from any cause without prior relapse are events assigned to the NRM with relapse as a competing event. OS was defined as the time from the date of cell transplantation to death or last contact. Median follow-up time was calculated using the reverse Kaplan-Meier method.

Neutrophil engraftment was defined as the first of three consecutive days with at least 500 granulocytes/µL after transplantation. Platelet engraftment was defined as the first platelet count greater than 20,000/µL for more than three days without platelet transfusion. Diagnosis and grading of acute GvHD (aGvHD) and chronic GvHD (cGvHD) were performed according to standard criteria [7–10]. Primary cause of post-transplant death was assessed according to the criteria of Copelan et al. [11].

### Statistical analysis

Patients were divided into beta-blocker users and non-users according to the definitions outlined above. Normality of continuous variables was assessed using the Shapiro–Wilk test. Two independent groups were compared using the Mann–Whitney U test for continuous variables and the χ² test (or Fisher’s exact test when appropriate) for categorical variables. OS was estimated with the Kaplan–Meier method and compared using the log-rank test. CIR and NRM were estimated using the Aalen–Johansen estimator. Survival probabilities were reported at 2 years. Univariate and multivariable effects were assessed for CIR and NRM using the Fine–Gray subdistribution hazards model, and for OS using Cox proportional hazards models. The following covariates were included a priori to control for confounding rather than selected by model-selection procedures: age, sex mismatch, donor match, donor relationship, remission status, and beta-blocker use.

The *P* value for interaction was obtained from likelihood-ratio tests comparing nested models with and without the interaction term. Analyses were conducted using RStudio (version 1.1.463) and SPSS (version 26). Statistical significance was defined as a two-sided *P* value < 0.05.

## Results

### Patient characteristics

A total of 413 patients with AML who underwent allogeneic HSCT met the eligibility criteria for this retrospective single-center study. At the time of the analysis, 183 (44.3%) patients had died. Patient, disease, and transplant characteristics are listed in Table 1.

**Table 1:** Patient, disease and transplantation characteristics

Variables	No beta-blocker	Beta-blocker	*P* value
N, no (%)	301 (72.9)	112 (27.1)	
Age, years			< 0.001*
Median (range)	52 (18–75)	59 (20–75)	
Sex, no (%)			0.117**
Male	170 (56.5)	73 (65.2)	
Female	131 (43.5)	39 (34.8)	
AML type, no (%)			0.072**
*de novo*	232 (77.1)	79 (70.5)	
t-AML	17 (5.6)	3 (2.7)	
s-AML	52 (17.3)	30 (26.8)	
ELN 2010, no (%)			0.433**
Favorable	52 (17.3)	18 (16.1)	
Intermediate	179 (59.5)	63 (56.3)	
Adverse	68 (22.6)	31 (27.7)	
Unknown	2 (0.7)	0 (0)	
NPM1 status, no (%)			0.792**
Wild-type	231 (76.7)	88 (78.6)	
Mutated	69 (22.9)	24 (21.4)	
Unknown	1 (0.3)	0 (0)	
FLT3 status, no (%)			0.453**
Wild-type	231 (77.0)	91 (81.3)	
ITD	61 (20.3)	17 (15.2)	
TKD	8 (2.7)	4 (3.6)	
Remission status at HSCT, no (%)			0.512**
Complete remission (CR)	165 (54.8)	55 (49.1)	
CR1	140 (46.5)	45 (40.2)	
CR ≥ 2	25 (8.3)	9 (8.0)	
not determinable	0	1 (0.9)	
Active AML	136 (45.2)	57 (50.9)	
Refractory	105 (34.9)	46 (41.1)	
Untreated relapse	31 (10.3)	11 (9.8)	
Comorbidities, no (%)			
Hypertension	65 (21.6)	58 (51.8)	< 0.001*
Atrial fibrillation/flutter	6 (2.0)	14 (12.5)	< 0.001*
Coronary heart disease	2 (0.7)	7 (6.3)	0.002**
Donor age, years			0.227*
Median (range)	37 (18–73)	39 (19–76)	
Donor type, no (%)			0.163**
Related Mismatched	1 (0.3)	1 (0.9)	
Related Matched	89 (29.6)	27 (24.1)	
Unrelated Matched	157 (52.2)	70 (62.5)	
Unrelated Mismatched	51 (16.9)	12 (10.7)	
Haploidentical	3 (1.0)	2 (1.8)	
Sex mismatch, no (%)			0.349**
Female D/male R	48 (15.9)	13 (11.6)	
Other	253 (84.1)	99 (88.4)	
Graft source, no (%)			1.000**
PBSC	291 (96.7)	108 (96.4)	
BM	10 (3.3)	4 (3.6)	
CMV risk, no (%)			0.701**
D-/R-	73 (24.3)	27 (24.1)	
D+/R-	32 (10.6)	12 (10.7)	
D-/R+	68 (22.6)	31 (27.7)	
D+/R+	128 (42.5)	42 (37.5)	
Conditioning, no (%)			0.247**
MAC	7 (2.3)	0 (0)	
RIC	135 (44.9)	44 (39.3)	
NMA	7 (2.3)	2 (1.8)	
SEQ	152 (50.5)	66 (58.9)	
ATG, no (%)			0.677**
Yes	241 (80.1)	92 (82.1)	
No	60 (19.9)	20 (17.9)	
Immunosuppression, no (%)			0.050**
CsA/MMF	163 (54.2)	66 (58.9)	
CsA/MTX	137 (45.5)	43 (38.4)	
Other	1 (0.3)	3 (2.7)	
Neutrophil Engraftment, days			0.002*
Median (range)	18 (7–38)	16 (8–28)	
Platelet Engraftment, days			0.820*
Median (range)	18 (7–84)	19 (3–83)	
Acute GvHD, no (%)			1.000**
Grade 0–1	239 (79.4)	89 (79.5)	
Grade ≥ 2	62 (20.6)	23 (20.5)	
Chronic GvHD, no (%)			0.101**
No cGvHD	184 (61.1)	70 (62.5)	
Mild	29 (9.6)	11 (9.8)	
Moderate	34 (11.3)	20 (17.9)	
Severe	54 (17.9)	11 (9.8)	
Primary Cause of Death, no (%)			0.041**
Relapse	48 (37.5)	10 (18.2)	
Graft failure	6 (4.7)	2 (3.6)	
aGvHD	8 (6.3)	1 (1.8)	
cGvHD	15 (11.7)	14 (25.5)	
Infection	34 (26.6)	15 (27.3)	
Organ Toxicity	6 (4.7)	5 (9.1)	
Other	6 (4.7)	3 (5.5)	
Unknown	5 (3.9)	5 (9.1)	

AML acute myeloid leukemia, ELN European Leukemia Net, CR complete remission, PBSC peripheral blood stem cells, BM bone marrow, D donor, R recipient, CMV cytomegalovirus, MAC myeloablative conditioning, RIC reduced intensity conditioning, NMA non-myeloablative conditioning, SEQ sequential conditioning, ATG antithymocyte globulin, CsA cyclosporine A, MMF mycophenolate mofetil, MTX methotrexate, GvHD graft-versus-host disease

Statistical analysis: * Mann-Whitney U; ** χ² (Fisher’s exact test when appropriate)

The beta-blocker group included 112 patients (27.1%). The control group consisted of 301 patients (72.9%) who were not taking beta-blockers according to the definitions as outlined above. The median (range) age of the entire study population was 54 (18–75) years. Beta-blocker users were older than patients in the control group (median 59 vs. 52 years, *P* < 0.01) and more frequently had pre-existing cardiovascular comorbidities, including hypertension, atrial fibrillation, and coronary heart disease (Table 1). Two hundred and eighteen patients (52.8%) received a sequential conditioning regimen (SEQ) and 179 patients (43.3%) received fludarabine and total body irradiation (4 × 2 Gy) or a combination of melphalan and fludarabine with treosulfan or busulfan (RIC). In addition, 9 patients (2.2%) received non-myeloablative conditioning regimens (NMA), mostly consisting of fludarabine and total body irradiation with 2 × 2 Gy. Seven patients (1.7%) received myeloablative conditioning regimens (MAC). A total of 399 patients (96.6%) received peripheral blood stem cells (PBSC) and 14 patients (3.4%) received a bone marrow (BM) graft. Of the 413 patients, 220 patients (53.3%) were in complete remission (CR) prior to transplantation and 193 patients (46.7%) had active disease. Cyclosporine A in combination with methotrexate for GvHD prophylaxis was used in 180 cases (43.6%). Cyclosporine A in combination with mycophenolate mofetil was given to 229 patients (55.4%), and 4 patients (1.0%) received other medications for GvHD prophylaxis.

Two hundred and twenty-four patients (54.2%) were treated with at least one of the following cardiac medications: 112 patients (27.1%) were taking beta-blockers, 138 patients (33.4%) were treated with calcium channel blockers, and 83 patients (20.1%) were taking ACE inhibitors or ARBs. The distribution of medication use is shown in Supplement 1. Use of ACE inhibitors, ARBs or calcium channel blockers was not significantly associated with outcome (all *P* > 0.05).

### Beta-blocker use and outcome after transplantation

**Fig. 2:**
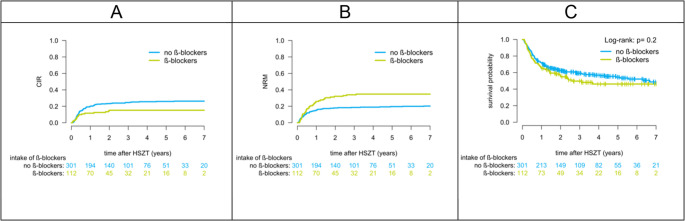
(**A**) Cumulative incidence of relapse (CIR), (**B**) non-relapse mortality (NRM), and (**C**) overall survival (OS) after HSCT in patients receiving or not receiving beta-blockers. *P* values were obtained using Gray’s test (A, B) and the log-rank test (C).

Median follow-up was 43.2 months (95% CI, 39.6–51.2). Median OS for the entire cohort was not reached during follow-up. At 2 years, CIR was lower in the beta-blocker group than in controls (16.0% vs. 25.4%; *P* = 0.024; Fig. 2a). Conversely, the cumulative incidence of NRM was higher with beta-blocker use (35.7% vs. 19.1%; *P* = 0.002; Fig. 2b). For OS, the estimated 2-year survival was 49.7% (95% CI, 39.8–59.6) with beta-blockers and 59.3% (95% CI, 53.5–65.0) without beta-blockers (*P* = 0.353; Fig. 2c).

**Fig. 3:**
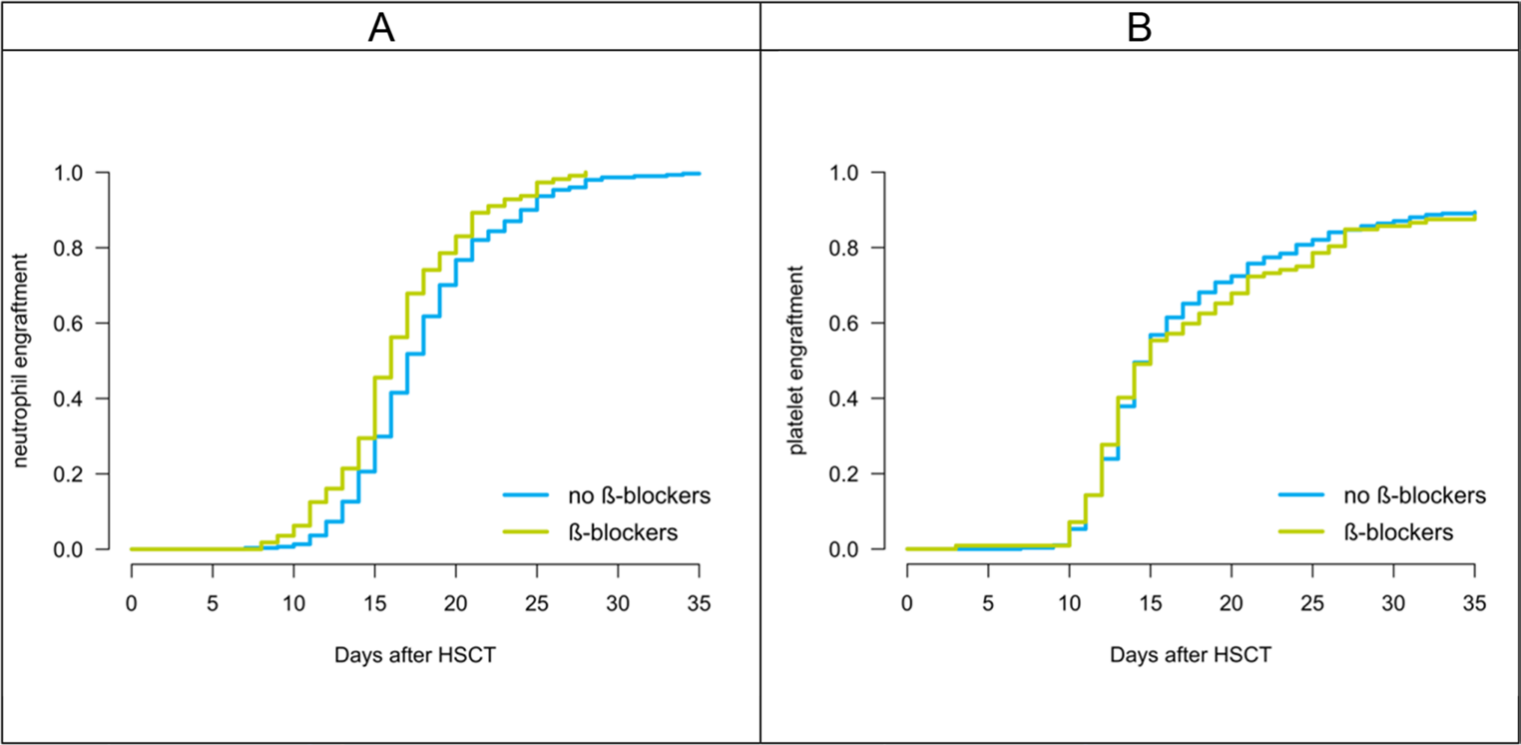
(**A**) Neutrophil and (**B**) platelet engraftment after HSCT in patients receiving or not receiving beta-blockers. *P* values were derived from log-rank tests.

Eighty-five patients (20.6%) experienced aGvHD ≥ grade 2 and 159 (38.5%) patients had cGvHD. The beta-blocker and control groups did not differ in the incidence of acute or chronic GvHD (*P* > 0.05). In the beta-blocker group, 89 patients (79.5%) had no or grade 1 aGvHD, and 23 patients (20.5%) had aGvHD grade 2 or higher. In the control group, 239 patients (79.4%) had no aGvHD or aGvHD grade 1, and 62 patients (20.6%) had aGvHD grade 2 or higher. cGvHD affected 42 patients (37.5%) in the beta-blocker group and 117 patients (38.9%) in the control group (Fig. 3).

**Fig. 4:**
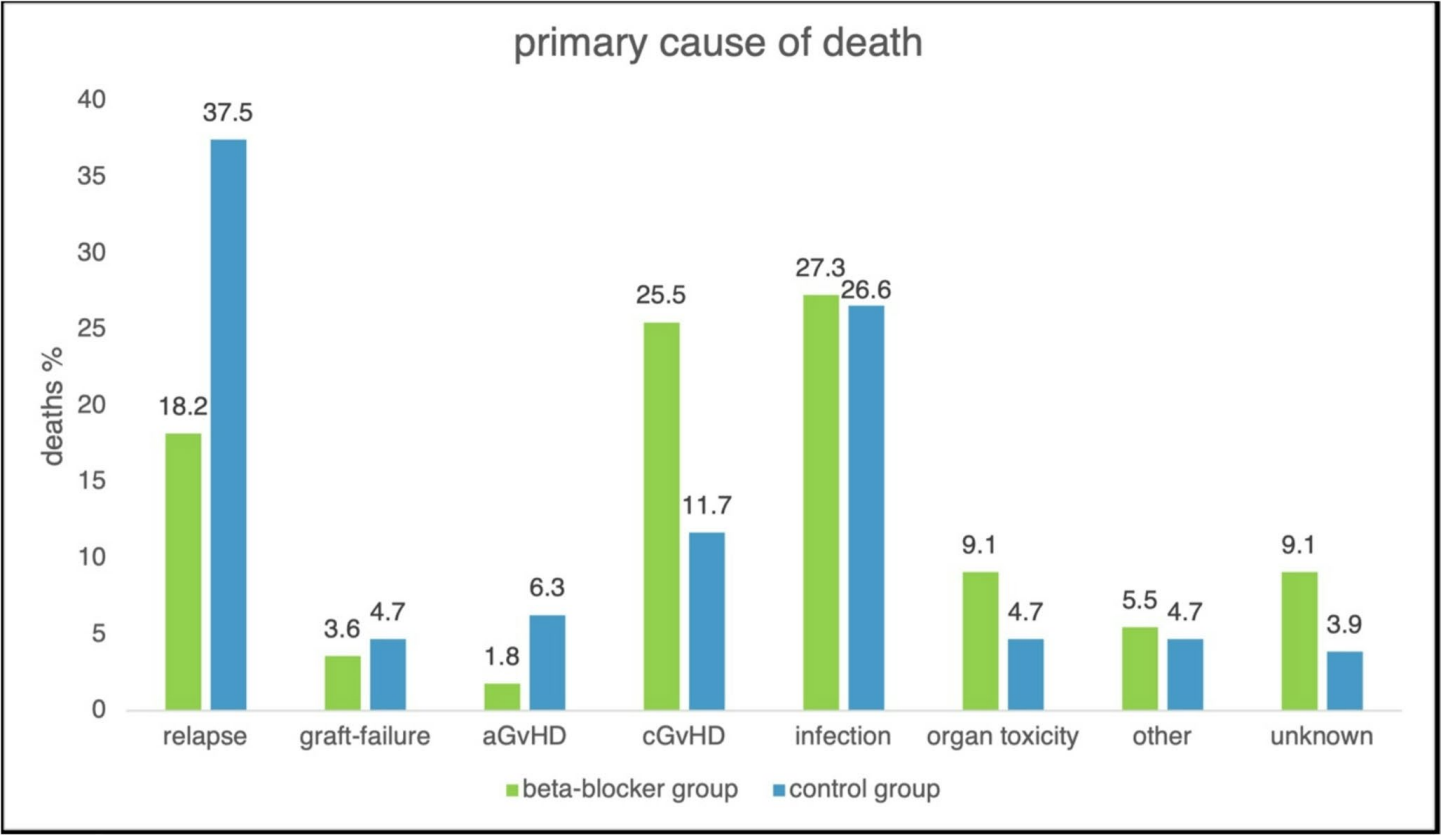
Distribution of primary causes of death, according to previously published definitions [11], in patients receiving beta-blockers (green) and control patients (blue). aGvHD = acute graft-versus-host disease; cGvHD = chronic graft-versus-host disease

The primary cause of death differed significantly between the beta-blocker and control groups (*P* = 0.041). In the beta-blocker cohort, death was attributed to infection in 15 (27.3%) patients, cGvHD in 14 (25.5%) and AML relapse in 10 (18.2%). In the control group, the main cause of death was AML relapse in 48 (37.5%) patients, followed by infection in 34 (26.6%) and cGvHD in 15 (11.7%). Differences in primary cause of death are shown in Fig. 4.

Univariate analyses of potential risk factors for CIR, NRM, and OS are shown in Table 2. Older age and active disease at transplantation were consistently associated with inferior outcomes across endpoints. For OS, additional associations were observed with donor match, donor relation, remission status, and conditioning therapy. Beta-blocker use was associated with a lower CIR but a higher NRM, while no significant association with OS was detected. Adjusted associations are reported in Table 3 and summarized below.

**Table 2:** Univariate analysis for cumulative incidence of relapse, non-relapse mortality and overall survival

Covariables	CIR		NRM		OS	
	Fine-Gray		Fine-Gray		Cox proportional hazards	
	HR (95% CI)	*P*	HR (95% CI)	*P*	HR (95% CI)	*P*
Age	0.99 (0.98–1.01)	0.620	1.02 (1.00-1.04)	0.018	1.14 (1.01–1.28)	0.032
Sex mismatch						
No	Ref		Ref		Ref	
Yes	0.86 (0.48–1.54)	0.620	1.07 (0.63–1.81)	0.800	0.98 (0.66–1.47)	0.923
Donor match						
Matched	Ref		Ref		Ref	
Mismatched	1.05 (0.62–1.76)	0.860	1.76 (1.10–2.82)	0.019	1.64 (1.15–2.34)	0.006
Donor relation						
Related	Ref		Ref		Ref	
Unrelated	1.41 (0.89–2.25)	0.150	1.23 (0.80–1.90)	0.340	1.45 (1.04–2.02)	0.030
Remission						
CR	Ref		Ref		Ref	
active disease	1.08 (0.73–1.62)	0.690	1.95 (1.31–2.91)	0.001	1.60 (1.20–2.15)	0.001
Beta-blocker						
Non-use	Ref		Ref		Ref	
Use	0.55 (0.33–0.94)	0.028	1.86 (1.25–2.77)	0.002	1.24 (0.90–1.70)	0.180

*Ref* Reference, *HR* Hazard ratio, *CIR* cumulative incidence of relapse, *NRM* non-relapse mortality, *OS* overall survival;

Age was modeled per 1-year increase for CIR and NRM, and per 10-year increase for OS

**Table 3:** Multivariable analysis for cumulative incidence of relapse, non-relapse mortality and overall survival

Covariables	CIR		NRM		OS	
	Fine-Gray		Fine-Gray		Cox proportional hazards	
	HR (95% CI)	*P*	HR (95% CI)	*P*	HR (95% CI)	*P*
Age (per 10 years)	1.00 (0.87–1.14)	0.960	1.15 (0.95–1.40)	0.160	1.09 (0.97–1.24)	0.157
Sex mismatch						
No	Ref		Ref		Ref	
Yes	0.94 (0.52–1.70)	0.830	1.12 (0.65–1.93)	0.680	1.06 (0.70–1.60)	0.786
Donor match						
Matched	Ref		Ref		Ref	
Mismatched	0.90 (0.52–1.56)	0.710	1.73 (1.09–2.74)	0.020	1.54 (1.08–2.22)	0.019
Donor relation						
Related	Ref		Ref		Ref	
Unrelated	1.45 (0.87–2.44)	0.160	1 (0.65–1.58)	0.970	1.27 (0.89–1.80)	0.181
Remission						
CR	Ref		Ref		Ref	
active disease	1.08 (0.72–1.63)	0.720	1.78 (1.19–2.67)	0.005	1.49 (1.11-2.00)	0.009
Beta-blocker						
Non-use	Ref		Ref		Ref	
Use	0.53 (0.31–0.92)	0.024	1.72 (1.13–2.64)	0.012	1.17 (0.84–1.62)	0.353

*Ref* Reference, *HR* Hazard ratio, *CIR* cumulative incidence of relapse, *NRM* non-relapse mortality, *OS* overall survival

In multivariable analyses, adjusting for demographics, donor characteristics and transplantation-related variables, beta-blocker use was independently associated with lower CIR (hazard ratio (HR), 0.53; 95% CI, 0.31–0.92; *P* = 0.024) and higher NRM (HR 1.72; 95% CI, 1.13–2.64; *P* = 0.012), but not OS (*P* = 0.353). Other variables associated with NRM were donor match (*P* = 0.020) and remission status before transplant (*P* = 0.005). There were no other covariates with an independent effect on CIR in our model. The results of the multivariable regression analysis are listed in Table 3.

Beta-blocker use was associated with an earlier neutrophil engraftment (at median on day 16 vs. 18 after HSCT in the beta-blocker group vs. control group; *P* = 0.002; Fig. 3a). There was no significant difference in platelet engraftment between the two groups (at median on day 19 vs. 18 after HSCT in the beta-blocker group vs. control group; *P* > 0.05; Fig. 3b).

In the beta-blocker group, 56 patients (50%) received bisoprolol and 51 patients (45.5%) received metoprolol. Only five patients (4.5%) were treated with other agents such as propranolol or carvedilol. Fifty-one patients (45.5%) received low-dose beta-blockers and 61 patients (54.5%) high-dose beta-blockers, according to the definitions given above. Medication characteristics are listed in Supplement 2. There was no difference in CIR, NRM and OS among high- and low-dose beta-blocker users (*P* > 0.05 for all outcome variables, data not shown). Similarly, there was no significant difference in CIR, NRM and OS between the different beta-blocker substances (*P* > 0.05).

The results of the subgroup analysis are shown in the forest plot for OS (Supplement 3). Exploratory analyses suggested significant interactions of beta-blocker use with antithymocyte globulin (ATG) administration (P_interaction = 0.01) and donor relation (P_interaction = 0.01). In patients transplanted from related donors, who uniformly did not receive ATG, beta-blocker use was associated with inferior OS (HR 2.58, 95% CI 1.42–4.67).

## Discussion

In our analysis, among AML patients who received a first allogeneic HSCT, the use of beta-blockers was associated with a lower risk of post-transplant AML relapse. In contrast, the risk of death without prior AML relapse was higher among beta-blocker users. As a net effect of the lower CIR but higher NRM in beta-blocker users, OS did not differ between the beta-blocker and the control groups. Since age alone does not seem to be the main driver of this observation according to the results of the multivariable analyses, it is likely that the variable “beta-blocker use” marks vulnerable patients with a higher burden of pre-existing medical conditions in our retrospective analysis. Consistent with this interpretation, patients receiving beta-blockers more frequently had pre-existing cardiovascular comorbidities, including hypertension, atrial fibrillation, and coronary heart disease. These comorbidities may at least partly explain the higher non-relapse mortality observed among beta-blocker users. In this context, the higher proportion of infection-related deaths among beta-blocker users should be interpreted with caution, as reduced physiological reserve in patients with cardiovascular comorbidities may contribute to fatal infectious complications after HSCT rather than a direct effect of beta-blockade. On the other hand, we also investigated whether the use of calcium channel blockers, ACE inhibitors or ARBs predicted post-transplant outcome and found no significant associations with CIR, NRM or OS. However, within the group of patients on cardiovascular medications, the need for beta-blockers may still label the more vulnerable fraction of patients, i.e. those with a history of angina, myocardial infarction, or atrial fibrillation, whereas calcium channel blockers, ACE inhibitors or ARBs may be used by patients with arterial hypertension only.

Nevertheless, beta-blocker users had a lower cumulative risk of AML relapse in our study after adjusting for demographic, disease- and transplantation-related confounders, which may possibly indicate improved immunologic control of residual leukemia after transplantation. However, a dose-dependent effect was not observed. While there was no difference between beta-blocker users and non-users in the incidence of acute or chronic GvHD, consistent with a potential immunomodulatory effect of beta-blockers, we found that cGvHD was a major cause of death among patients in the beta-blocker group, ranking second after infections. In contrast, patients in the control group died mostly due to AML relapse.

In exploratory subgroup analyses, beta-blocker use showed a significant association with outcome among patients who either did not receive antithymocyte globulin (ATG) or underwent transplantation from a related donor. As related donor recipients in our cohort uniformly did not receive ATG, these factors were closely intertwined. Given the exploratory nature of these findings, they should be interpreted with caution. Our study has several limitations, most of which are inherent to its retrospective nature. First, we applied an engraftment-landmark design; patients who died before neutrophil engraftment were excluded. Consequently, causes of very early mortality, including early infectious deaths, could not be assessed. This may introduce selection bias and prevents assessment of very early post-transplant mortality. In addition, although patient characteristics such as age differed between the beta-blocker and non-beta-blocker groups, we cannot rule out additional unmeasured confounders. It also remains difficult to disentangle the relative contributions of underlying cardiovascular conditions from potential immune-related mechanisms to post-transplant NRM. Second, we were not able to demonstrate a dose–response relationship between beta-blocker dose and outcome variables. Third, beta-blocker exposure beyond our predefined criteria could not be verified in individual patients. Finally, although non-selective beta-blockers such as propranolol may exert stronger immunomodulatory effects than β1-selective agents the small number of patients receiving non-selective agents precluded further analysis [12]. Despite these limitations, our study is the first to demonstrate an association between beta-blocker use and post-transplant outcome in a large cohort of AML patients undergoing allogeneic HSCT. If confirmed in independent cohorts and laboratory studies, our findings may provide a rationale to investigate the effects of carefully titrated beta-adrenergic blockade on donor cell-mediated immune responses and post-transplant outcome in a prospective trial.

## Supplementary Information

Below is the link to the electronic supplementary material.

Supplementary Material 1

## Data Availability

The datasets generated and/or analyzed during the current study are not publicly available due to data protection and ethical restrictions but are available from the corresponding author on reasonable request.
