# Supplementary material for: Beta-blocker use and outcome after allogeneic hematopoietic stem cell transplantation in acute myeloid leukemia
Source: Ann Hematol. 2026 Mar 25;105(4):205. doi: 10.1007/s00277-026-06962-w (PMC13013103; doi:10.1007/s00277-026-06962-w)
Supplement: Supplementary file 1 — Supplementary Material 1 [file 277_2026_6962_MOESM1_ESM.docx]

**Supplement 1. Use of cardiovascular medication.**

Venn diagram showing the distribution of patients treated with beta-blockers, calcium channel blockers, and ACE inhibitors or angiotensin II receptor blockers (ARBs).Values indicate the proportion of patients (%) in each subgroup.

12%

9.8%

7.6%

11.2%

**Supplement 2. Medication characteristics.**

Baseline characteristics according to the use of beta-blockers, calcium channel blockers, and ACE inhibitors or angiotensin II receptor blockers (ARBs). Data are shown as n (%) unless otherwise specified. P values were obtained by χ² test for categorical variables and Mann–Whitney U test for continuous variables.

| Medication characteristics | | | | |
| --- | --- | --- | --- | --- |
| **Variables** | **control group** | **beta-blocker group** | **p-value** | **total** |
| **Beta-blocker**, n (%) | 301 (72.9) | 112 (27.1) |  |  |
| **Substance beta-blocker**, no (%) |  |  |  |  |
| Bisoprolol |  | 56 (50.0) |  |  |
| Metoprolol |  | 51 (45.5) |  |  |
| Other |  | 5 (4.5) |  |  |
| **Dose beta-blocker**, no (%) |  |  |  |  |
| low |  | 51 (45.5) |  |  |
| high |  | 61 (54.5) |  |  |
| **ACE inhibitors**, no (%) | 29 (9.6) | 30 (26.8) | 0.001 | 59 (14.3) |
| **Calcium channel blockers** , no (%) | 95 (31.6) | 43 (38.4) | 0.199 | 138 (33.4) |
| **Angiotensin II receptor blockers**, no (%) | 10 (3.3) | 15 (13.4) | 0.001 | 25 (6.1) |
| **ACE inhibitors and/or angiotensin II receptor blockers**, no (%) | 39 (13.0) | 44 (39.3) | 0.001 | 83 (20.1) |

**Supplement 3.** **Forest plot for OS**.

Treatment effect on OS in subgroups. Vertical line represents the hazard ratio (HR) of the total cohort. P for interaction was obtained from likelihood-ratio tests of models with vs. without the interaction term.

**
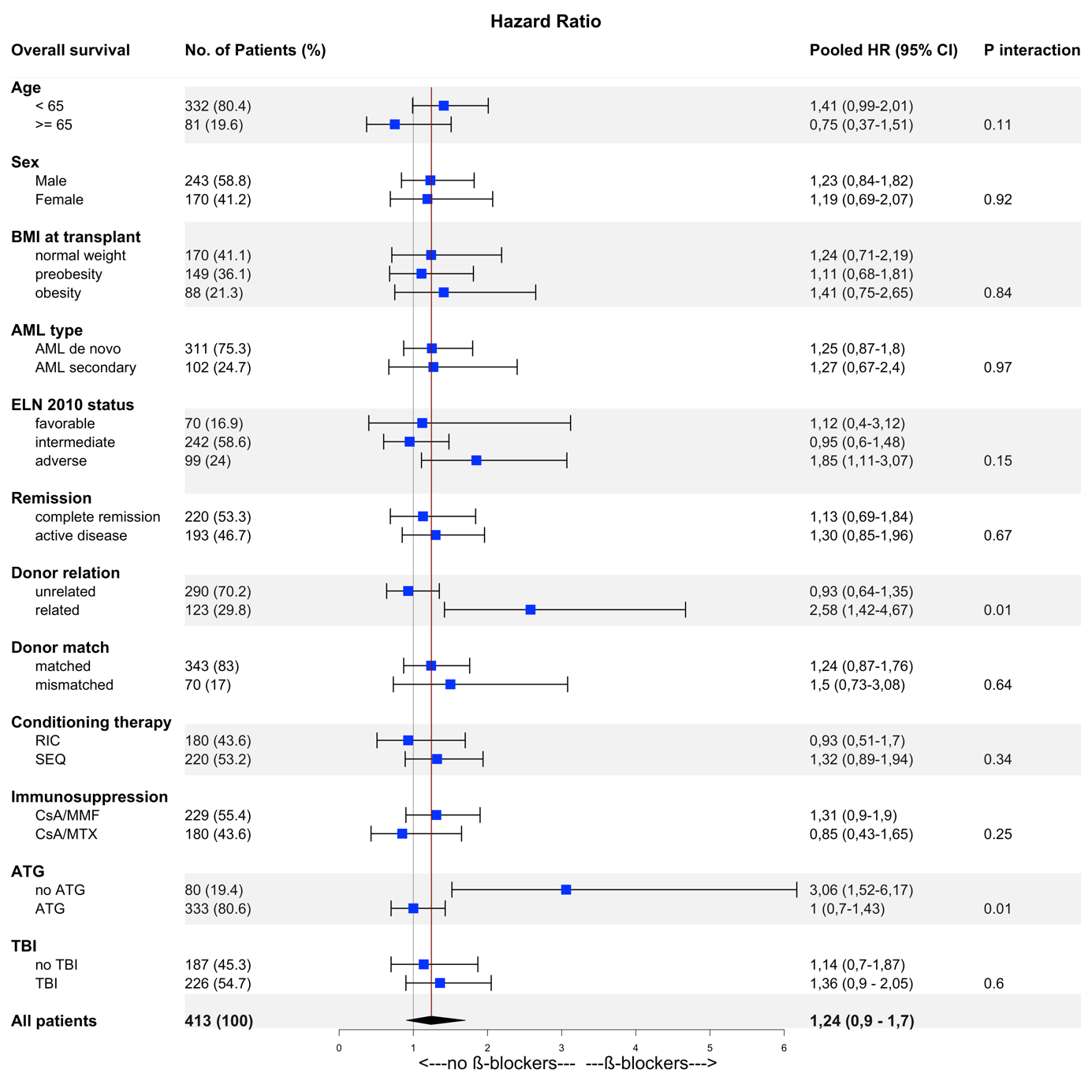
**

**Supplement 4. Distribution of beta-blocker use by year of HSCT**

| **Year of HSCT** | **control group, n** | **beta-blocker group, n** |
| --- | --- | --- |
| 2011 | 35 | 5 |
| 2012 | 43 | 17 |
| 2013 | 34 | 11 |
| 2014 | 34 | 13 |
| 2015 | 40 | 16 |
| 2016 | 39 | 18 |
| 2017 | 47 | 19 |
| 2018 | 29 | 13 |

Distribution of beta-blocker use and non-use according to the year of HSCT.
Values represent absolute numbers of patients per year.
